# Non-invasive estimation of the parameters of a three-element windkessel model of aortic arch arteries in patients undergoing thoracic endovascular aortic repair

**DOI:** 10.3389/fbioe.2023.1127855

**Published:** 2023-02-28

**Authors:** Rosamaria Tricarico, Scott A. Berceli, Roger Tran-Son-Tay, Yong He

**Affiliations:** ^1^ Department of Biomedical Engineering, University of Florida, Gainesville, FL, United States; ^2^ Division of Vascular Surgery and Endovascular Therapy, Department of Surgery, University of Florida, Gainesville, FL, United States; ^3^ North Florida/South Georgia Veterans Health System, Gainesville, FL, United States; ^4^ Department of Mechanical and Aerospace Engineering, University of Florida, Gainesville, FL, United States

**Keywords:** image-based computational fluid dynamics, thoracic endovascular aortic repair, three-element windkessel model, boundary conditions, personalized cardiovascular medicine

## Abstract

**Background:** Image-based computational hemodynamic modeling and simulations are important for personalized diagnosis and treatment of cardiovascular diseases. However, the required patient-specific boundary conditions are often not available and need to be estimated.

**Methods:** We propose a pipeline for estimating the parameters of the popular three-element Windkessel (WK3) models (a proximal resistor in series with a parallel combination of a distal resistor and a capacitor) of the aortic arch arteries in patients receiving thoracic endovascular aortic repair of aneurysms. Pre-operative and post-operative 1-week duplex ultrasound scans were performed to obtain blood flow rates, and intra-operative pressure measurements were also performed invasively using a pressure transducer pre- and post-stent graft deployment in arch arteries. The patient-specific WK3 model parameters were derived from the flow rate and pressure waveforms using an optimization algorithm reducing the error between simulated and measured pressure data. The resistors were normalized by total resistance, and the capacitor was normalized by total resistance and heart rate. The normalized WK3 parameters can be combined with readily available vessel diameter, brachial blood pressure, and heart rate data to estimate WK3 parameters of other patients non-invasively.

**Results:** Ten patients were studied. The medians (interquartile range) of the normalized proximal resistor, distal resistor, and capacitor parameters are 0.10 (0.07–0.15), 0.90 (0.84–0.93), and 0.46 (0.33–0.58), respectively, for common carotid artery; 0.03 (0.02–0.04), 0.97 (0.96–0.98), and 1.91 (1.63–2.26) for subclavian artery; 0.18 (0.08–0.41), 0.82 (0.59–0.92), and 0.47 (0.32–0.85) for vertebral artery. The estimated pressure showed fairly high tolerance to patient-specific inlet flow rate waveforms using the WK3 parameters estimated from the medians of the normalized parameters.

**Conclusion:** When patient-specific outflow boundary conditions are not available, our proposed pipeline can be used to estimate the WK3 parameters of arch arteries.

## 1 Introduction

Thoracic endovascular aortic repair (TEVAR) has been increasingly used to treat aortic arch pathologies (Wallen et al., 2018; Brown et al., 2021). However, in contrast to other locations along the aortic tree, aortic arch endografts are subjected to more severe biomechanical forces that can lead to post-operative complications (Scali et al., 2012; Pecoraro et al., 2017; Voskresensky et al., 2017). In this perspective, computational fluid-dynamics (CFD) simulations have contributed to the investigation of the mechanisms of aortic stent graft complications following TEVAR (Gallo et al., 2016; Madhavan and Kemmerling, 2018; van Bakel et al., 2018; Tricarico et al., 2020b; Hu et al., 2022). Since its first biomedical applications at the end of the 20th century (Stergiopulos et al., 1992), eased by the evolution and better accessibility of medical imaging tools and computational resources, computational modeling has been widely utilized to investigate hemodynamic characteristics that are difficult to measure *in vivo* (Sengupta et al., 2022). The ongoing optimization of these tools and their regulation aim to their safe integration into the biomedical device investigation for personalized treatment, which will be fundamental to the development of next-generation cardiovascular devices.

However, among the major challenges of patient-specific computational modeling is obtaining the patient-specific input data, which directly influence result accuracy, but often are not available (Morris et al., 2016; Gray and Pathmanathan, 2018; Madhavan and Kemmerling, 2018; He et al., 2022). In the absence of patient-specific measurements, the state of the art of boundary condition estimation for CFD analysis is using data from healthy subjects reported in the literature (Lantz et al., 1981; Taylor et al., 1998; Olufsen et al., 2000) and often integrated into lumped parameter Windkessel models (Armour et al., 2022). The Windkessel model (Westerhof et al., 2009) uses electrical analogues to describe a hydraulic system, where pressure (P) and flow (Q) are analogous to voltage and current respectively (Garber et al., 2022). When patient-specific flow rate and/or pressure waveforms are not available, a Windkessel model is a common strategy to impose vascular outlet boundary conditions; it has the advantage of allowing for interdependent time-varying flow rate and pressure distributions. The most popular Windkessel model has three elements, a proximal resistor (R_1_) in series with a parallel combination of a distal resistor (R_2_) and a capacitor (C). These parameters represent the total resistances (R_tot_, R_1_ + R_2_) and compliances distal to the artery of interest, receiving the arterial flow rate under a specific pressure. Previous studies have demonstrated that, compared with zero-pressure assumption at the outlets, a three-element Windkessel (WK3) model achieves a better overall performance in terms of matching the inflow data and producing physiological pressure waveforms (Morbiducci et al., 2010; Pirola et al., 2017).

In this study, we analyzed ultrasound-derived patient-specific flow rate and intra-operatively measured pressure waveforms to build a set of algorithms for first-degree estimation of the parameters of the WK3 model that can be utilized when patient-specific flow rate and pressure waveforms are not available. The WK3 parameters can be estimated using more readily available patient-specific data, such as vessel diameter that can be extracted from computed tomography angiography (CTA) and brachial artery pressure that can be measured non-invasively by a cuff.

## 2 Materials and methods

### 2.1 Parameter estimation pipeline

**FIGURE 1:**
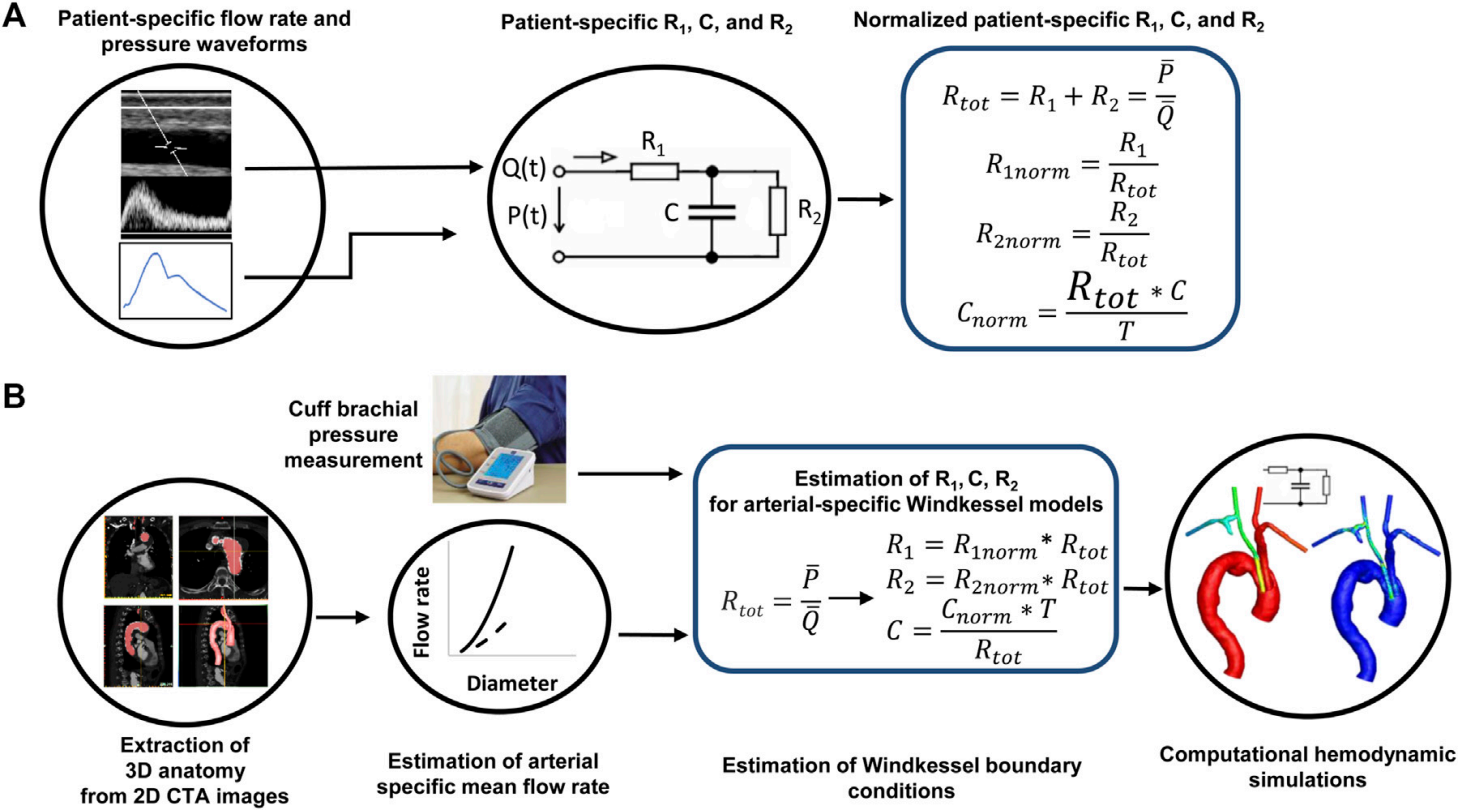
The pipeline of patient-specific estimation of the parameters of a three-element Windkessel model when lacking patient-specific flow rate and pressure waveforms. **(A)** Derivation of normalized Windkessel model parameters from patient-specific flow rate and pressure waveforms. **(B)** Estimation of Windkessel model parameters informed by non-invasive vascular imaging and pressure data. R_tot_: the sum of the two resistances; T: the cardiac period.

We propose a pipeline for estimating the parameters of the WK3 model of the aortic arch branch arteries when the artery-specific flow rate and pressure waveforms are not available (Figure 1). The goal is to use the artery-specific total resistance (R_tot_) and cardiac period (T) to scale the population-averaged, artery-specific normalized R_1_, C, and R_2_ values (R_1nom_, C_norm_, and R_2norm_) to estimate the non-normalized parameters as:

$$R_{1}={R_{1norm}∙R}_{tot};{\;R}_{2}={R_{2norm}∙R}_{tot};\;C=\frac{C_{norm}∙T}{R_{tot}}$$ (1)

To encompass the patient-to-patient variability of mean flow rates and pressures, normalized R_1_, C, and R_2_ parameters are used and defined as:

$$R_{1norm}=\frac{R_{1}}{R_{tot}};\;R_{2norm}=\frac{R_{2}}{R_{tot}}=1-R_{1norm};\;C_{norm}=R_{tot}∙\frac{C}{T}$$ (2)

T_tot_ is calculate from mean pressure ( $\bar{P}$ ) and mean flow rate ( $\bar{Q}$ ) as described in Eq. 3.

$$R_{tot}=R_{1}+R_{2}=\frac{\bar{P}}{\bar{Q}};$$ (3)

The mean arterial pressure can be estimated from the non-invasively measured systolic (SBP) and diastolic (DBP) brachial blood pressures *via* a commonly used equation

$$\bar{P}=\frac{1}{3}\left(SBP-DBP\right)+DBP$$ (4)

assuming systole is one-third of the cardiac cycle (Sesso et al., 2000). Mean flow rate can be measured by Doppler ultrasound if available or estimated from the previously published flow rate-diameter relationships based on the diameter extracted from CTA images, such as those reported by us (Tricarico et al., 2020a). CTA is routinely performed for diagnosis and treatment planning of aortic pathologies and is often used in clinical research to extract three dimensional arterial models. Cardiac period can be calculated from heart rate. Furthermore, by defining the normalized flow rate, pressure, and time as each variable divided by its mean (flow rate, pressure) and T, respectively, the differential equation governing the relation between flow rate and pressure in the WK3 model is maintained (Supplementary Material). The population-averaged, artery-specific R_1norm_, C_norm_, and R_2norm_ values can be extracted from measured flow rate and pressure data. We describe the extraction and assessment of the combinations of normalized parameters in the next sections.

### 2.2 Patient-specific flow rate and pressure data acquisition and processing

We developed a prospective study to measure patient-specific waveforms of flow rate and pressure data in aortic arch branch arteries in 10 patients suffering aortic aneurysms or dissections, undergoing TEVAR. The protocol for this prospective study was approved by the University of Florida College of Medicine Institutional Review Board (Gainesville, FL, United States), and informed consent was signed by every patient. Duplex ultrasound measurements were acquired under resting conditions (with awake patients in supine position) using a Philips iU22 system. Measurements were collected on multiple locations of bilateral common carotid (proximal, middle, and distal), subclavian (distal to the vertebral artery, thyrocervical and costocervical trunks, and internal thoracic artery), and vertebral arteries at pre-operative and 1-week post-operative time in the vascular laboratory. We did not take intra-operative flow rate measurements as this would interrupt the standard TEVAR procedure. Arterial diameters and flow rates were extracted from ultrasound images. Details on the methodology of flow rate calculation have been described in our previous work (Tricarico et al., 2020a).

Intra-operative pressure measurements were performed invasively using a TruWave disposable pressure transducer (Edwards Lifesciences, Irvine, CA, United States) before and after stent graft deployment in brachiocephalic artery, left common carotid, and left subclavian arteries, all carried out after catheter flushing. All pressure waveforms were traced and smoothed in Matlab R2017b (Mathworks Inc., Natick, MA, United States) using the heart rate extracted from ultrasound images, due to patient sedation at the time of the intra-operative pressure measurements. In addition, non-invasive brachial artery pressures were collected by a cuff at the time of the ultrasound imaging and mean arterial pressure was estimated using Eq. 4.

### 2.3 Patient-specific R_1_, C, and R_2_ identification

We identified the non-normalized R_1_, C, and R_2_ values for each artery (common carotid, subclavian, and vertebral arteries) of the patients to obtain the average of each parameter for this patient cohort using the Simulink Design Optimization toolbox (Mathworks Inc.), where the WK3 model was built, and the governing equation of the WK3 model is embedded into the block diagram (additional details in Supplementary Figure S1). Measured patient-specific, pre-operative flow and pre-deployment pressure waveforms (referred to as training dataset in Section 2.5) were used as imposed input and expected output of the model, respectively. Since there were no intra-operative pressure measurements in the right common carotid artery and subclavian artery, pressure waveforms measured at the corresponding left side arteries were utilized for simulations on the right vasculature. In addition, subclavian artery pressures were used for vertebral artery simulations, due to invasiveness and risks of intra-deployment measurements in vertebral arteries.

Because R_tot_ = R_1_+R_2_, there are only two independent parameters in the WK3 model that need to be identified. The mean flow rate and mean pressure used to calculate R_tot_, were obtained from the corresponding waveforms. The Runge-Kutta method was chosen as the solver with a fixed time step of 10^–4^ s to maximize the accuracy of pressure waveform calculation iteratively. A non-linear least square method and trust-region-reflective algorithm were utilized with both parameter tolerance and function tolerance of 0.001. The sum of squared error (SSE) between measured and simulated pressure of the WK3 model was chosen as the cost function for the optimization problem. At each iteration, the cost function quantified the quality of the pressure matching, and at the end of the optimization process, the optimized R_1_, C, and R_2_ combination was collected. Additionally, the L^2^-norm of the relative error for pressure estimation in time was calculated as in Eq. 5 and collected for each R_1_, C, and R_2_ combination on each artery.

$${\left|\left|e\right|\right|}_{L^{2}}=\sqrt{\frac{\sum_{k=1}^{N}{\left|P_{simul}\left(t_{k}\right)-P_{\exp}\left(t_{k}\right)\right|}^{2}}{\sum_{k=1}^{N}P_{\exp}{\left(t_{k}\right)}^{2}}*100}$$ (5)

where P_simul_ and P_exp_ are simulated and experimentally measured pressures, respectively; N is the number of time steps in a cardiac period. Additional details of the parameter estimation process are given in Supplementary Figure S2. After obtaining the optimized, non-normalized R_1_, C, and R_2_ values for each artery, the corresponding normalized R_1_, C, and R_2_ values were calculated as Eq. 2.

### 2.4 Sensitivity analysis of the predicted pressure waveform on normalized R_1_, C, and R_2_ parameters

A Monte Carlo method was performed in the Simulink Design Optimization Toolbox to examine the sensitivity of predicted pressure to the parameters of the WK3 model. For purposes of comparison, all flow rate and pressure waveforms were normalized to their means in Matlab. In addition, time-normalization by the cardiac period and interpolation (every 0.01) were performed. For each type of artery, the averages of the normalized pre-operative flow rate and pre-deployment pressure waveforms were imposed respectively as input and expected output of the WK3 model. Accordingly, normalized WK3 model parameters were used as the input. 1500 sets of the two independent parameters (R_1norm_ and C_norm_) were randomly generated under the hypothesis of a uniform distribution for the three arterial-specific Monte Carlo analyses. The simulation ran until a minimum cost function was achieved.

### 2.5 Assessment of the normalized R_1_, C, and R_2_

The normalized R_1_, C, and R_2_ values obtained from pre-operative flow and pre-deployment pressure data (Section 2.3, training dataset) were assessed using the post-operative flow at 1 week and post-deployment pressure data (testing dataset) for the left arteries of all patients. Specifically, the medians of the normalized R_1_, C, and R_2_ obtained from the training dataset were scaled to each $R_{tot}$ and T to estimate the patient-specific, non-normalized R_1_, C, and R_2_ parameters of each artery, as detailed in Eq. 1. R_tot_ was calculated from the mean flow rate and pressure calculated from the measured waveforms of the testing dataset. Each estimated R_1_, C, and R_2_ and patient-specific (non-normalized) input flow rate were used in the Winkessel model to estimate pressure waveforms using Simulink Design Optimization toolbox. Relative errors between estimated and measured artery-specific pressure waveforms were calculated. Additionally, the estimated R_1_, C, and R_2_ values were tested on the Windkessel model, imposing non-normalized pressure waveforms as input and flow rate waveforms as expected output, to quantify relative errors between estimated and measured flow rate waveforms. Simulated pressure and flow rate waveforms are presented in normalized form for comparison purposes.

### 2.6 Statistical analysis

Both non-normalized and normalized parameter values are presented as artery-specific median and 25th-75th percentiles (interquartile range, IQR). Other data were presented as mean ± standard deviation. T-tests or Mann-Whitney rank sum tests were performed to detect statistical differences in flow rate and pressure means. Statistical analyses were performed in Sigmaplot (SYSTAT Software Inc., Chicago, IL, United States). A *p*-value <0.05 was considered statistically different.

## 3 Results

### 3.1 Patient cohort

The analyzed population, 10 patients with age 64 ± 3 (range, 40–82) years and body surface area 2.0 ± 0.3 (range, 1.6–2.3) m^2^, was 50% male. Four of ten patients underwent percutaneous transluminal repair only, either with TEVAR isolated to the descending thoracic aorta or TEVAR with a fenestrated branch to the left subclavian artery. The remaining six patients underwent a hybrid TEVAR procedure, which involved left subclavian artery coverage and a left common carotid artery-left subclavian artery bypass. The average heart rate for these patients was 63 ± 10 beats per minute (range, 47–76 beats per minute). The most common comorbidities for this set of subjects are listed in Table 1. The majority of subjects suffered hypertension (80%) and were active or former tobacco users (60%).

**TABLE 1:** Percentage of patients with comorbidities.

Hypertension (HTN)	80 (%)
Active/former tobacco users	60%
Hyperlipidemia (HDL)	50%
Previous aneurysm interventions	40%
Congestive heart failure (CHF)	20%
Cerebral artery disease	20%
Arrhythmia	20%
Chronic obstructive pulmonary disease (COPD)	10%
Gastroesophageal reflux disease (GERD)	10%
Carotid artery disease	10%
Sleep apnea	10%
Arthritis	10%

### 3.2 Flow rate and pressure data

No statistically significant difference of the mean flow rate was observed between the right and left sides. Therefore, the main results are presented on left and right arteries combined hereafter. The mean flow rates of the pre-operative dataset for common carotid artery (458 ± 139 mL/min), subclavian artery (185 ± 116 mL/min) and vertebral artery (90 ± 59 mL/min) were not statistically different from the means of the post-operative 1 week dataset (428 ± 147, 228 ± 116, and 92 ± 48 mL/min for common carotid, subclavian, and vertebral arteries, respectively). No significant differences of the mean pressures between left common carotid artery and subclavian artery of the pre-deployment (83 ± 11 vs. 85 ± 12 mmHg for common carotid artery and subclavian artery, respectively) or post-deployment (89 ± 13 vs. 88 ± 10 mmHg for common carotid and subclavian arteries, respectively) dataset were observed, neither between pre- and post-deployment mean pressures in each artery.

**FIGURE 2:**
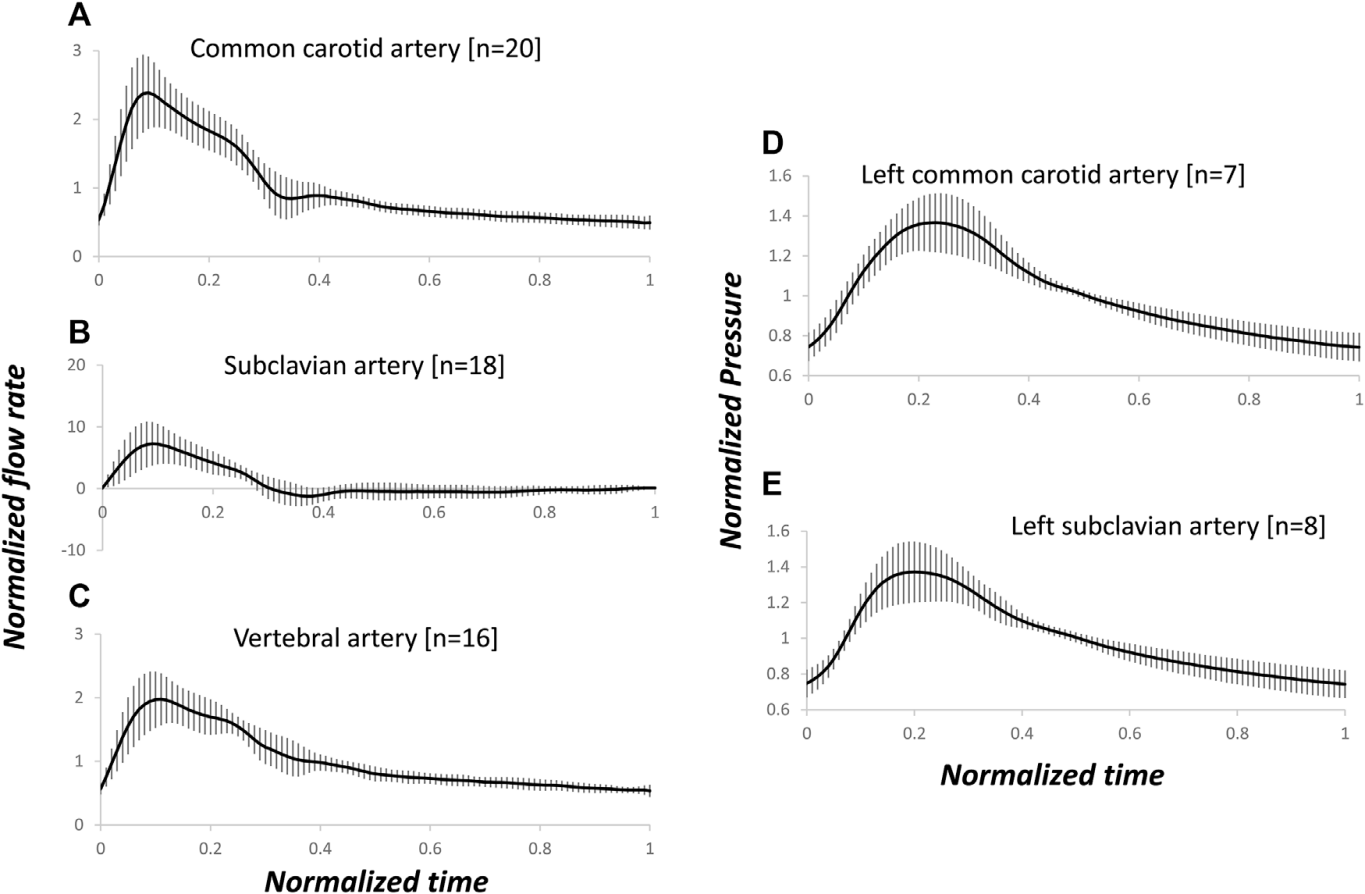
Arterial-specific, pre-operative, normalized flow rate and pre-deployment, normalized pressure waveforms. The flow rate and pressure were normalized by the corresponding mean of each waveform. Average and standard deviation of flow rates in common carotid **(A)**, subclavian **(B)** and vertebral **(C)** arteries, and pressure in left common carotid **(D)** and subclavian **(E)** arteries. The number of measured blood vessels, n, is also shown.

Figures 2A–C present the patient-specific normalized arterial flow rate waveforms for the pre-operative dataset. Notably, the subclavian artery showed systolic high peak and reversal flow at early diastole (Zhang et al., 2022), not present in the mono-phasic waveforms of common carotid artery and vertebral artery. Figures 2D, E present the normalized, pre-deployment pressure waveforms in the left common carotid artery and subclavian artery.

### 3.3 Patient-specific R_1_, C, and R_2_ parameters

**FIGURE 3:**
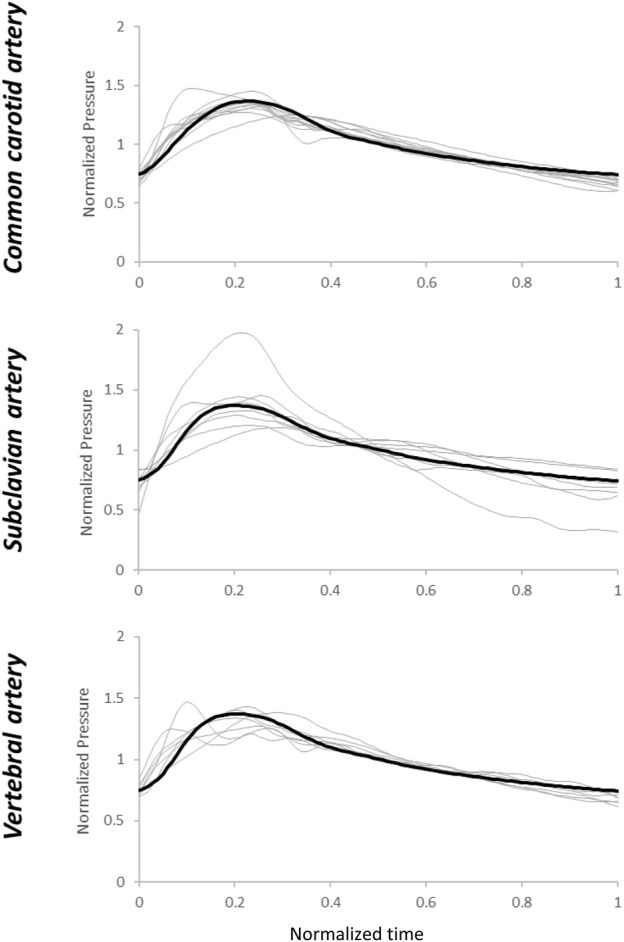
Estimated pressure waveforms using patient-specific, pre-operative input flow rate waveforms. The black line represents the average of the measured waveforms. Normalization has been performed post-simulation for comparison purposes.

When the patient-specific optimized R_1_, C, and R_2_ parameters were obtained from the WK3 simulation (as described in Section 2.3), the relative errors on the pressure waveform matching were small and 4 (3–4) %, 4 (3–6) %, and 3 (3–5) % for common carotid, subclavian, and vertebral arteries, respectively (example of pressure waveform matching and corresponding relative error in Supplementary Figure S3). The individual estimated and average of measured pressure waveforms are shown in Figure 3. The patient-specific non-normalized and normalized parameters of the WK3 model for each type of artery are shown in Table 2.

**TABLE 2:** Arterial-specific normalized and non-normalized R_1_, C, and R_2_ parameters of common carotid, subclavian, and vertebral arteries from the ten analyzed patients.

*Artery*	*Non-Normalized Results*			*Normalized Results*		
	R_1_	R_2_	C	Normalized R_1_	Normalized R_2_	Normalized C
	[mmHg sec/mL]	[mmHg sec/mL]	[mL/mmHg]			
*Carotid arteries*	1.14 (0.72; 1.85)	10.35 (7.79; 12.45)	0.04 (0.02; 0.06)	0.10 (0.07; 0.15)	0.90 (0.84; 0.93)	0.46 (0.33; 0.58)
*Subclavian arteries*	0.96 (0.81; 1.42)	33.10 (18.99; 68.19)	0.06 (0.03; 0.11)	0.03 (0.02; 0.04)	0.97 (0.96; 0.98)	1.91 (1.63; 2.26)
*Vertebral arteries*	8.93 (6.71; 20.33)	37.79 (21.47; 92.04)	0.01 (0.00; 0.02)	0.18 (0.08; 0.41)	0.82 (0.59; 0.92)	0.47 (0.32; 0.85)

**FIGURE 4:**
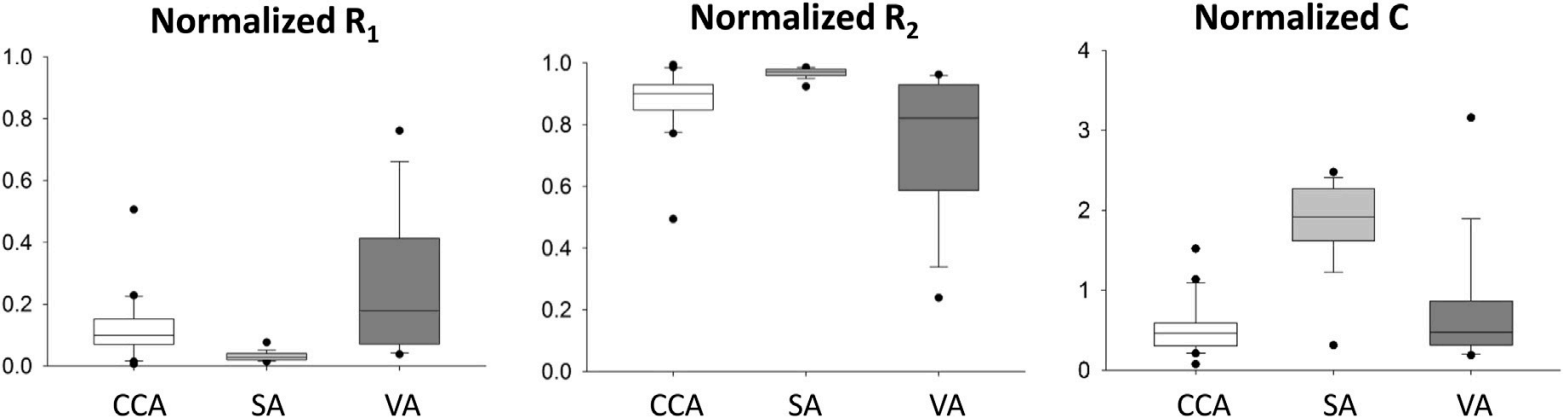
Distribution of patient-specific, normalized R_1_, C, and R_2_ parameters for common carotid (CCA), subclavian (SA), and vertebral (VA) arteries. In these plots, the minimum represents the data point with the lowest value above the first quartile minus 1.5 times of the interquartile range, and the maximum represents the data point with the highest value below the third quartile plus 1.5 times of the interquartile range. The lower vertical line connects the minimum to the first quartile; the upper vertical line connects the third quartile to the maximum.

The normalized WK3 model parameters are also shown in Figure 4 as boxplots. The fairly large ranges of normalized resistances and compliance on each artery can be related to the apparent variation of the normalized waveform profiles among patients (Figure 2).

### 3.4 Sensitivity analysis

**FIGURE 5:**
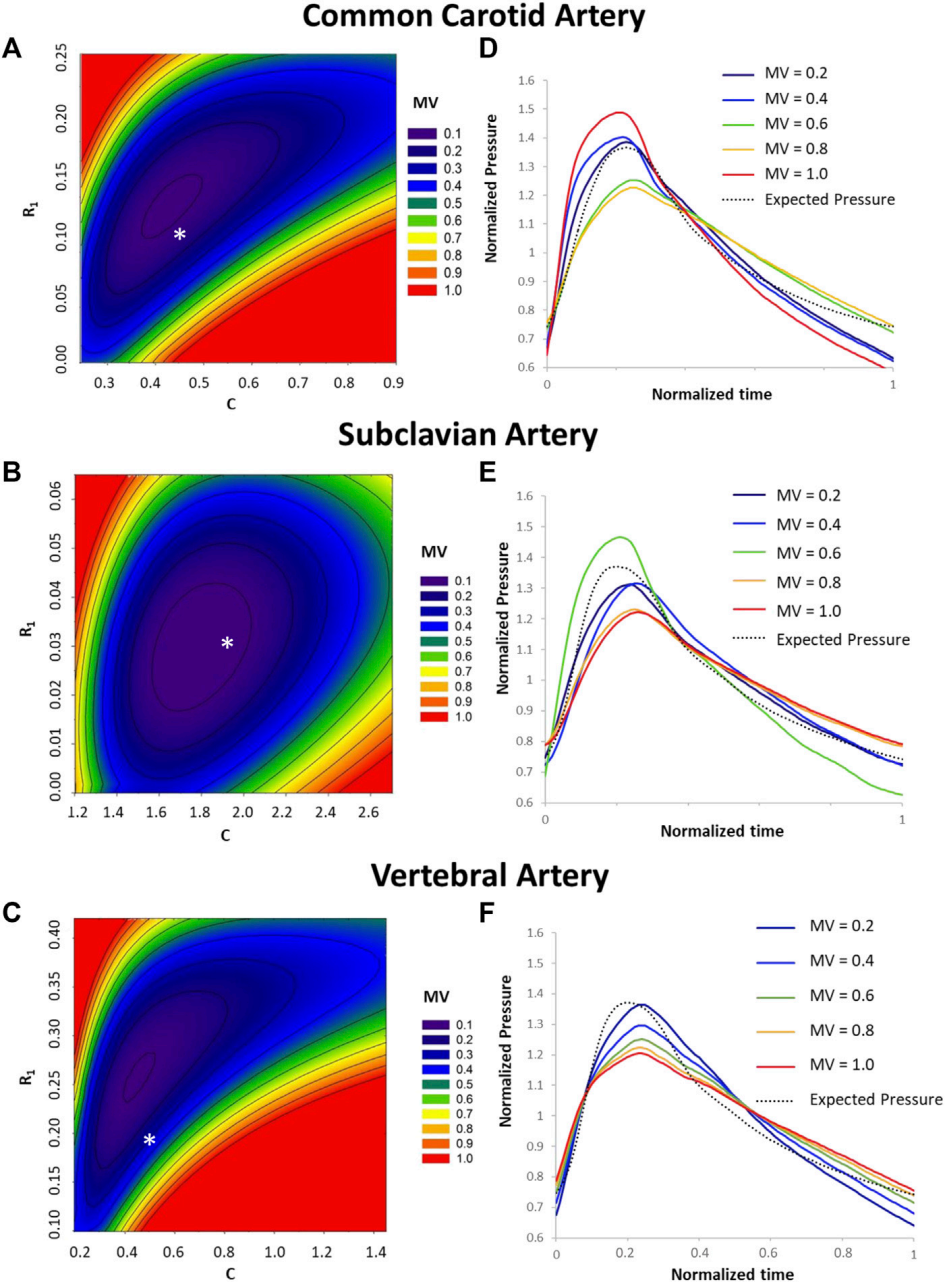
Sensitivity analysis of predicted pressure on variations of R_1_, C, and R_2_. Contour plot of minimized values (MV) under different combinations of the R_1_ and C values are shown for common carotid artery **(A)**, subclavian artery **(B)**, and vertebral artery **(C)**. For reference, the medians of normalized R_1_ and C values for each type of artery (Table 2) are shown by an * in A, B, and **(C)**. The minimized value is one of the outputs from the Optimization toolbox and quantifies the difference between the predicted and expected pressure values under a set of R_1_, C, and R_2_ values. A smaller minimized value corresponds to a better overall match between predicted and expected pressures. Plots of the normalized pressure waveforms under some minimized values are shown for common carotid artery **(D)**, subclavian artery **(E)** and vertebral artery **(F)**.

The Monte Carlo analysis provided information on the sensitivity of the pressure waveform to the variation of the R_1_, C, and R_2_ parameters for each type of artery. From the contour plots describing the output quality (minimized values) with different parameter combinations, we can see that the sensitivity of the pressure output to the WK3 parameters varies with different combinations of these parameters (Figure 5). For example, when C equals 0.5, the common carotid artery pressure is more sensitive to R_1_ when R_1_ is small (R_1_ < 0.05), and less sensitive when R_1_ is larger (Figure 5A). The combination of the medians of normalized R_1_ and C parameters obtained from WK3 simulations for both common carotid artery and subclavian artery falls into the area of minimized value equal to 0.1, indicating good matching between expected and simulated pressure waveform (Figures 5A, B). The combination of the medians of normalized parameters for the vertebral artery falls into the area of minimized value equal to 0.3, suggesting the existence of alternative R_1_ and C combinations which could better represent the pressure outlet waveform (Figure 5C). For all three arteries, the pressure output is more sensitive to variations of R_1_ than C (smaller R_1_ ranges than C ranges). Around the medians of the normalized parameters, the common carotid artery pressure is not sensitive to relatively small deviations of R_1_ and C. The pressure of subclavian artery is even more tolerable to deviations of C from their corresponding medians (Figure 5B). The contour plot of vertebral artery is similar to that of carotid artery (Figure 5C), although with different axes’ ranges. Around the medians of the normalized parameters, the vertebral artery pressure is more sensitive to small deviations of R_1_ and C for smaller R_1_ and larger C. Plots of the normalized pressure waveforms under some minimized values are shown for common carotid, subclavian, and vertebral arteries (Figures 5D–F). Note that the simulated pressure waveform may be different for the same minimized value although the overall differences are the same.

### 3.5 Assessment of the normalized R_1_, C, and R_2_ parameters

**FIGURE 6:**
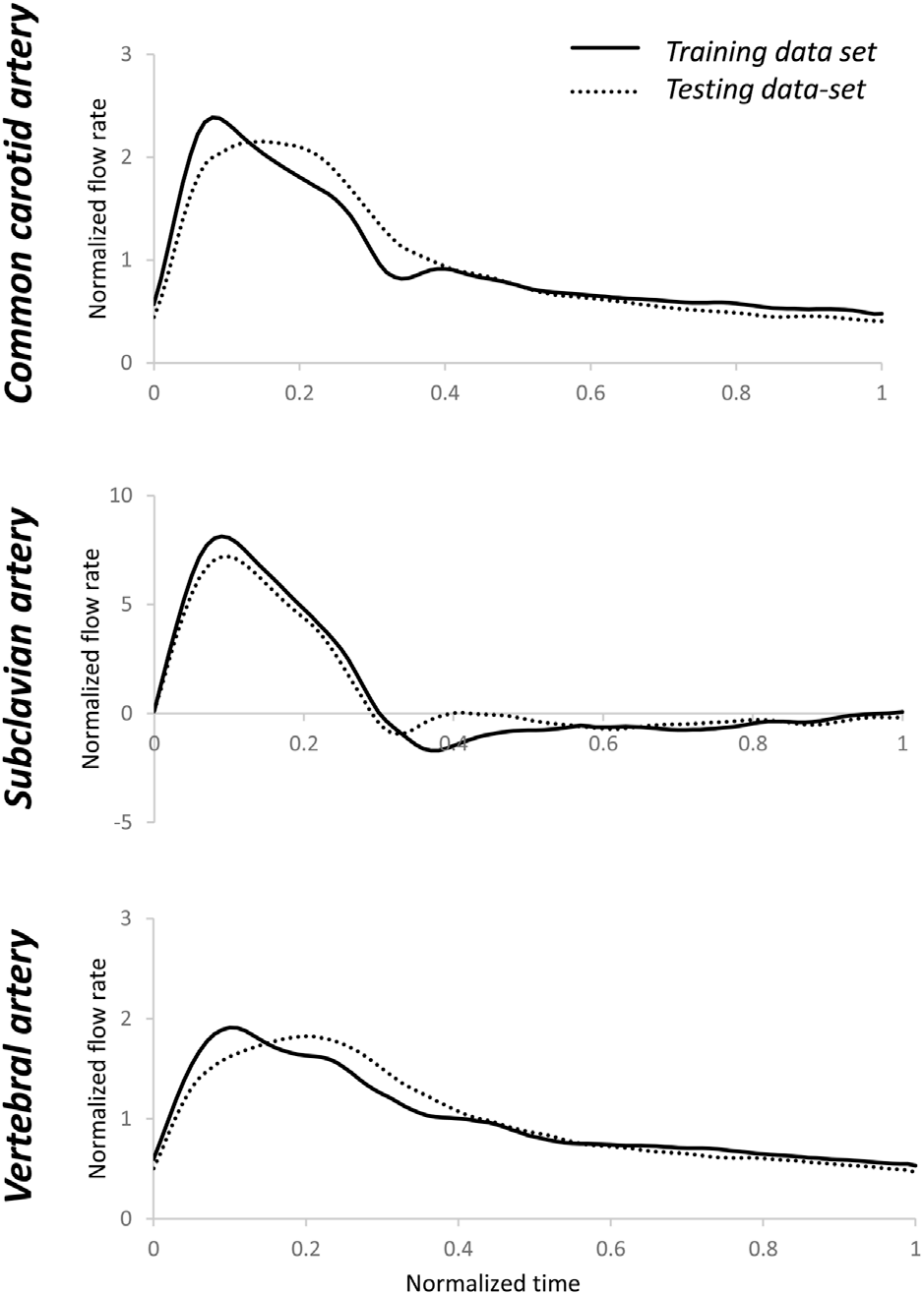
Averages of normalized testing flow rate waveforms for common carotid, subclavian, and vertebral arteries. The averages of the training flow rate waveforms that were used in section 2.3 are also shown. There were visible differences between the training and testing flow rate waveforms. Normalization was performed post-simulation for comparison purposes.

**FIGURE 7:**
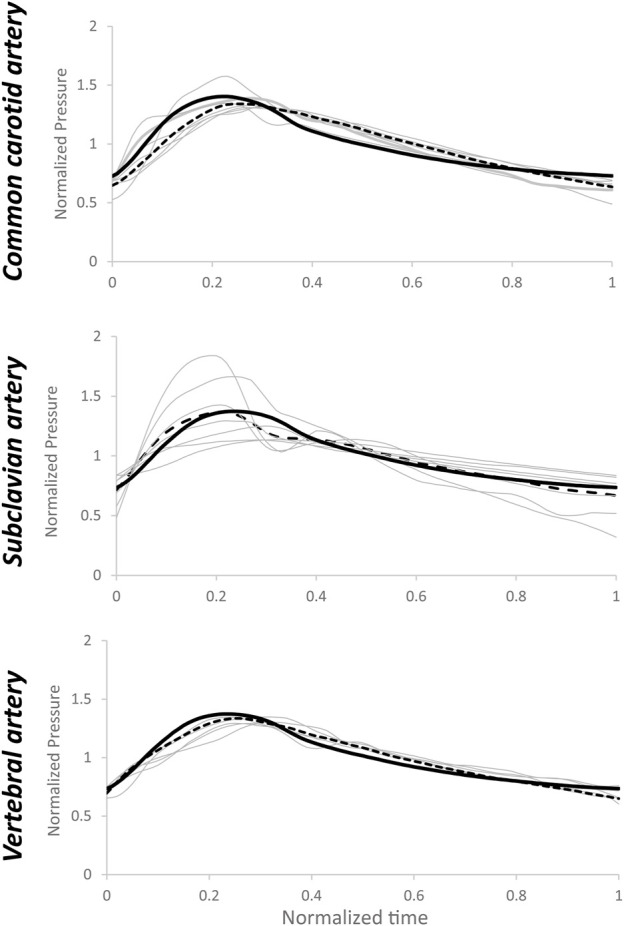
Estimated pressure waveforms (grey lines) using patient-specific inputs of flow rate data and estimated R_1_, C, and R_2_ values based on medians of arterial-specific parameters. Dotted lines represent the average of estimated pressure, while the black line represents the average value of measured pressure. Normalization has been performed post-simulation for comparison purposes.

**FIGURE 8:**
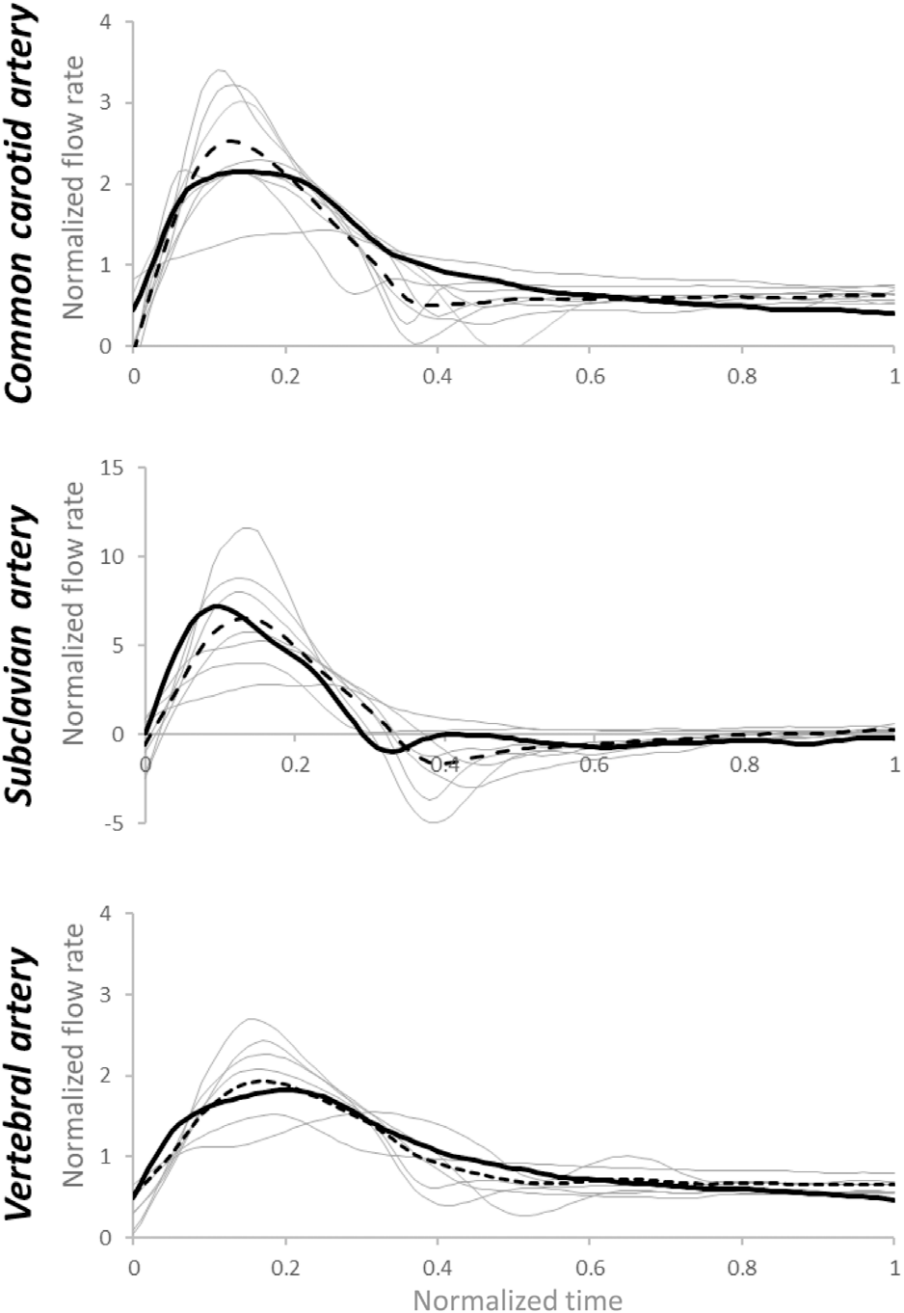
Estimated flow rate waveforms (grey lines) using patient-specific inputs of pressure data and estimated R_1_, C, and R_2_values based on medians of arterial-specific parameters. Dotted lines represent the average of estimated flow rates, while the black continuous line represents the average of measured flow rates. Normalization has been performed post-simulation for comparison purposes.

First, the individual flow rate waveform was used as the testing input, and the average flow rate waveform of the testing dataset for each type of artery is reported in Figure 6. The estimated pressure showed fairly high tolerance to patient-specific inlet flow rate waveforms using the estimated R_1_, C, and R_2_ parameters. Relative errors of pressure output of the testing dataset were: 13 (9–15) % for common carotid artery, 16 (10–17) % for subclavian artery, and 12 (11–16) % for vertebral artery. The estimated pressure waveforms are shown in Figure 7. Compared with the average of the measured waveforms, only two subclavian arteries presented largely out-of-range estimated pressure waveforms at the systolic peaks. An example of the measured and corresponding estimated pressure waveforms is shown in Supplementary Figure S4. These resulted from flow rate waveforms with very high peak systolic and large reversal diastolic flow. Using the pressure waveform as input, the relative errors of estimated flow rate waveform matching were higher: 34 (29–43) % for common carotid artery, 94 (74–116) % for subclavian artery, 30 (23–31) % for vertebral artery (Figure 8). One example of the measured and estimated flow rate waveforms of a subclavian artery using the measured pressure waveform as the input is shown in Supplementary Figure S5.

## 4 Discussion

When physiological pressure in the flow field is desired, the Windkessel model is one of the most commonly used methods for specifying the outlet boundary condition if the pressure waveforms at the outlets are not available. Based on patient-specific flow rate and pressure waveform measurements, we obtained the R_1_, C, and R_2_ parameters of the three elements of the Windkessel model of aortic arch arteries in patients undergoing thoracic endovascular aortic repair. These parameters were then normalized. The normalized parameters can be used to estimate the patient-informed, non-normalized parameters when only limited but common clinical data of the cohort of these patients are available. We have demonstrated that a reasonable pressure waveform could be obtained using estimated parameters of the Windkessel model.

The patient-specific R_1_, C, and R_2_ values of the arch arteries obtained in our study are in the order of magnitude of most R_1_, C, and R_2_ values of previously published studies (Table 3) (Alimohammadi et al., 2014; van Bakel et al., 2018; Bonfanti et al., 2019; Pirola et al., 2019; Armour et al., 2022). Differences in ranges are related to flow rate and pressure averages as well as Windkessel model settings, such as the steady-state conditions on the definition of R_tot_, which is not always imposed on the Windkessel model allowing larger ranges for the two, therefore independent, resistances. To the best of our knowledge, our study is the only one that uses artery-specific flow rate and pressure waveforms to identify the R_1_, C, and R_2_ values. Restricted by the setup in the operating room, recording pressure waveform requires additional dedicated devices, which might be the reason of only minimal and maximal pressure data were available in some studies (Alimohammadi et al., 2014; Bonfanti et al., 2019).

**TABLE 3:** The R_1_, C, and R_2_ parameters extracted from the literature.

References	R_1_ (mmHg sec/mL)	R_2_ (mmHg sec/mL)	C (mL/mmHg)
Common carotid artery			
Alimohammadi et al. (2014)	0.110	14.590	0.085
Bonfanti et al. (2019)	0.728–1.793	12.271–30.221	0.017–0.065
Pirola et al. (2019)	2.176	34.884	0.040
Armour et al. (2022)	0.675–3.301	10.353–21.005	0.080–0.147
Subclavian artery			
Alimohammadi et al. (2014)	0.150	11.410	0.110
van Bakel et al. (2018)	1.388	17.108	0.020
Bonfanti et al. (2019)	0.556–1.038	9.379–20.406	0.029–0.052
Pirola et al. (2019)	0.900	21.005	0.080
Armour et al. (2022)	0.450–0.975	3.451–12.453	0.133–0.427
Vertebral artery			
van Bakel et al. (2018)	3.713	40.615	0.01

A few strategies have been applied to estimate the parameters of Windkessel model when the flow rate and/or pressure waveforms are not available. When the flow rate waveform was available, but the pressure waveform was not available, a method was proposed to scale a baseline pressure waveform from the literature using the measured brachial mean and pulse pressures (Romarowski et al., 2018). They have demonstrated that a multivariable optimization approach based on available patient-specific phase-contrast magnetic resonant imaging (PC-MRI) data of flow rate waveform, similar to our patient-specific estimation of R_1_, C, and R_2_, provides the most similar results to patient-specific PC-MRI-observed hemodynamics. When none of the waveforms was available, iterative CFD simulations of dissected aorta were performed to minimize the differences between invasively measured minimal and maximal pressure values and the respective simulated values at the outlets by tuning R_1_, C, and R_2_. This process is time consuming and can take 8 h (Alimohammadi et al., 2014). In a later report by the same group, a fixed ratio of R_1_ to R_tot_ was set at the arch arteries, and the compliance attributed to all the outlets was distributed proportionally to the mean flow at each outlet (Bonfanti et al., 2019). Even though the pressure waveforms seem to be recorded, they were not used; instead, mean pressure values and MRI-derived flow spits at the branches were used to calibrate the Windkessel model parameters (Armour et al., 2022). R_1_ has been estimated using the artery lumen area and pulse wave velocity, which is also related to the arterial radius (Pirola et al., 2017). Another notable example of deriving the Windkessel model parameters when only the geometry is available is to use impedance in the frequency domain (Xu et al., 2018). The flow rate waveform at the branch was obtained by scaling the inlet flow rate waveform per Murray’s law of an empirical exponent in the range 2–3. Combining the pressure waveform obtained from the literature, Fourier transform of the flow rate and pressure waveforms was used to obtain the impedance. The parameters R_1_, R_2_, and C were calibrated to match the landmark values of the impedance. The advantage of this strategy is that an iterative optimization process is not needed once the flow rate and pressure waveforms are available. A recent study did not use any patient-specific information to estimate the parameters (Fatma et al., 2022). Instead, a pressure waveform was obtained from the literature; a series of CFD simulations were performed to update the flow rate and pressure waveforms at the outlets, which were then used to optimize the Windkessel model parameters in Matlab. Different from our current study using the whole pressure waveform, the sum of the differences in maximal, mean, and minimal pressures between expected and computed values was used as the objective function in the genetic optimization algorithm.

We did not obtain flow and pressure data at the inlet of ascending aorta and the outlet of descending aorta. When patient-specific data are not available, a few approaches have been developed to apply these boundary conditions. It is common to use an MRI-measured flow rate waveform from the literature but interpolate to the heart rate of the patient (Alimohammadi et al., 2014). A more complicated way considers the compliance of the aorta by introducing a capacitor before the aortic inlet of the 3D model. The flow rate entering the 3D model is determined by a lumped parameter model that receives a flow rate waveform obtained from the literature but adopted by the patient-specific hemodynamic data (cardiac output, heart rate, and systolic-to-diastolic duration ratio extracted from Doppler ultrasonography) (Bonfanti et al., 2019). The distal outlets use WK3 models (van Bakel et al., 2018; Bonfanti et al., 2019).

A Windkessel model represents the impedance of the distal vascular bed to blood flow (Westerhof et al., 2009). Cardiac output has been estimated from invasively or non-invasively measured peripheral pressure pulses using a WK3 model at the ascending aorta (De Wilde et al., 2007; Bogert et al., 2010). In this method, only the systolic portion of the arterial pressure curve was used, avoiding the issue of reversed flow during the diastole. We also tested the normalized R_1_, C, and R_2_ parameters using the pressure waveform as input. The relative errors of estimated flow rate waveform matching were higher than the relative errors of estimated pressure waveform matching when the flow rate waveform was the input, especially for the subclavian artery, which might have a very high peak systolic flow and a high reversed flow during diastole. However, as shown in Figures 2D, E, the normalized pressure waveforms of the left common carotid artery and left subclavian artery are hardly distinguishable although the flow waveforms of these two arteries are clearly different (Figures 2A, B). Therefore, flow rate waveforms are more informative than the pressure waveforms. These flow rate waveforms with a higher complexity cannot be adequately reproduced in detail from pressure waveforms by a simple WK3 model. A more complex model, such as a four-element WK model with an inertance term, might be able to capture more details of the waveforms and reduce the errors between measured and predicted flow rates (Stergiopulos et al., 1999). However, since the inertance is difficult to be estimated (Westerhof et al., 2009), we recommend the use of pressure waveform as the input to the WK3 model.

We suggest that when measured mean flow rate is not available, it can be estimated from published flow rate-diameter relationships. Different power laws have described the relationship between flow rate and diameter at various arterial levels (Murray, 1926; Cheng et al., 2007; Cebral et al., 2008; Revellin et al., 2009; Chnafa et al., 2017). We investigated the flow rate and diameter relationships of the arch branch arteries in the TEVAR patients using ultrasound-measured flow rates and CTA-measured lumen diameters (Tricarico et al., 2020a). The power values of the best fit flow rate-diameter relationships are between 1.6 and 2.4. However, for subclavian artery, the *R* ^2^ of the flow rate-diameter fit was only 0.20 due to the wide scatter of the flow rate-diameter points. This is actually not accidental because these TEVAR patients commonly have a pathological subclavian artery with a wide arrange of diameter sizes. A larger study is needed to define the flow rate-diameter relationships according to the patient and arterial pathological characteristics.

The medians of normalized R1 and C values for each type of artery (Table 2) did not fall in the exact minimum of the minimized value space (indicated with an *, Figure 5). In the sensitivity analysis, the averages of the normalized pre-operative flow rate and pre-deployment pressure were used. To fully evaluate the sensitivity of the estimated pressure on the WK3 parameters, we intentionally used a wide range of combinations of random WK3 parameters. Therefore, it is not a surprise to see that the medians of the patient-specific normalized parameters did not locate at the position with a minimum minimized value for certain artery, such as vertebral artery. But even for vertebral artery, the medians of the normalized parameters were at a position with a small minimized value.

There are limitations to the current study. We have demonstrated that a physiological pressure waveform can be generated from input flow rate waveform. The relative errors are due to a multitude of factors, including the reduction of patient-specific waveform variability to one artery-specific waveform profile, the simplistic nature of the lumped element model, and the small patient dataset. The availability of other information, such as the flow rate waveform of the internal carotid artery considering the patient gender, age, and cardiovascular disease state (Durka et al., 2018), will help reduce the relative error. The assumption of pressure symmetry was theoretically supported by the arterial connection at the cerebrovascular level (Circle of Willis) and confirmed by the small pressure difference on bilateral measurements of one patient (data not shown). However, it may not be valid for pathological cases and/or patients with interrupted circle of Willis (observed in 4%–16% of analyzed populations (Fawcett and Blachford, 1905; Iqbal, 2013; Klimek-Piotrowska et al., 2016). Moreover, subclavian artery pressures were used for vertebral artery simulations. We believe that such differences are minor to the scope of the study. Negative systolic peaks (reversal flow rates) such as those that occur in cases of subclavian steal syndrome, cannot be reproduced with the provided R_1_, C, and R_2_ parameters. Last, differences generated by the proposed framework and other methodology for boundary condition estimation for CFD applications have not been investigated. For these reasons, this methodology is of consideration for first-degree approximation of clinically-relevant hemodynamic waveforms.

In conclusion, based on the analysis of flow rate and pressure measurements in ten patients undergoing TEVAR procedures, we provided the R_1_, C, and R_2_ values of the arch arteries, which can be used directly in other CFD simulations when there are not any patient-specific data available. We also propose a pipeline to estimate R_1_, C, and R_2_ parameters for common carotid, subclavian, and vertebral arteries, based on brachial pressure values and mean flow rate estimated from arterial diameter, in case of lacking flow rate and/or pressure waveforms. In our pipeline, approximation of resistances (R_1_ and R_2_) and compliance (C) of a WK3 model is realized by multiplying and dividing, respectively, the provided values of normalized resistances and compliances parameters to the value of R_tot_. We also provide the variations of the WK3 model parameters, which can be used to quantify unavoidable uncertainties in hemodynamics when assumptions are made. This pilot study deserves future developments. A larger patient cohort is needed for a better population stratification (separating males from females, young from elderly, *etc.*). The flow rate-diameter relationship of these arteries, especially subclavian artery, can also be improved by larger studies.

## Data Availability

The raw data supporting the conclusions of this article will be made available by the authors, without undue reservation.

## References

[B1] Alimohammadi M. Agu O. Balabani S. Diaz-Zuccarini V. (2014). Development of a patient-specific simulation tool to analyse aortic dissections: Assessment of mixed patient-specific flow and pressure boundary conditions. Med. Eng. Phys. 36 (3), 275–284. 10.1016/j.medengphy.2013.11.003 24290844

[B2] Armour C. H. Guo B. Saitta S. Pirola S. Liu Y. Dong Z. (2022). Evaluation and verification of patient-specific modelling of type B aortic dissection. Comput. Biol. Med. 140, 105053. 10.1016/j.compbiomed.2021.105053 34847383

[B3] Bogert L. W. J. Wesseling K. H. Schraa O. Van Lieshout E. J. De Mol B. A. J. M. Van Goudoever J. (2010). Pulse contour cardiac output derived from non-invasive arterial pressure in cardiovascular disease. Anaesthesia 65 (11), 1119–1125. 10.1111/j.1365-2044.2010.06511.x 20860647

[B4] Bonfanti M. Franzetti G. Maritati G. Homer-Vanniasinkam S. Balabani S. Díaz-Zuccarini V. (2019). Patient-specific haemodynamic simulations of complex aortic dissections informed by commonly available clinical datasets. Med. Eng. Phys. 71, 45–55. 10.1016/j.medengphy.2019.06.012 31257054

[B5] Brown J. A. Arnaoutakis G. J. Szeto W. Y. Serna-Gallegos D. Sultan I. (2021). Endovascular repair of the aortic arch: State of the art. J. Cardiac Surg. 36 (11), 4292–4300. 10.1111/jocs.15920 34405439

[B6] Cebral J. R. Castro M. A. Putman C. M. Alperin N. (2008). Flow-area relationship in internal carotid and vertebral arteries. Physiol. Meas. 29 (5), 585–594. 10.1088/0967-3334/29/5/005 18460763 PMC2692290

[B7] Cheng C. Helderman F. Tempel D. Segers D. Hierck B. Poelmann R. (2007). Large variations in absolute wall shear stress levels within one species and between species. Atherosclerosis 195 (2), 225–235. 10.1016/j.atherosclerosis.2006.11.019 17169362

[B8] Chnafa C. Bouillot P. Brina O. Delattre B. M. A. Vargas M. I. Lovblad K. O. (2017). Vessel calibre and flow splitting relationships at the internal carotid artery terminal bifurcation. Physiol. Meas. 38 (11), 2044–2057. 10.1088/1361-6579/aa92bf 29019794

[B9] De Wilde R. B. P. Schreuder J. J. Van Den Berg P. C. M. Jansen J. R. C. (2007). An evaluation of cardiac output by five arterial pulse contour techniques during cardiac surgery. Anaesthesia 62 (8), 760–768. 10.1111/j.1365-2044.2007.05135.x 17635422

[B10] Durka M. J. Wong I. H. Kallmes D. F. Pasalic D. Mut F. Jagani M. (2018). A data-driven approach for addressing the lack of flow waveform data in studies of cerebral arterial flow in older adults. Physiol. Meas. 39 (1), 015006. 10.1088/1361-6579/aa9f46 29205172 PMC5811231

[B11] Fatma K. Carine G.-C. Marine G. Philippe P. Valérie D. (2022). Numerical modeling of residual type B aortic dissection: Longitudinal analysis of favorable and unfavorable evolution. Med. Biol. Eng. Comput. 60 (3), 769–783. 10.1007/s11517-021-02480-1 35076858

[B12] Fawcett E. Blachford J. V. (1905). The circle of willis: An examination of 700 specimens. J. Anat. physiology 40 (1), 63–70. PMC1287340 17232664

[B13] Gallo D. Lefieux A. Morganti S. Veneziani A. Reali A. Auricchio F. (2016). A patient-specific follow up study of the impact of thoracic endovascular repair (TEVAR) on aortic anatomy and on post-operative hemodynamics. Comput. Fluids 141, 54–61. 10.1016/j.compfluid.2016.04.025

[B14] Garber L. Khodaei S. Keshavarz-Motamed Z. (2022). The critical role of lumped parameter models in patient-specific cardiovascular simulations. Archives Comput. Methods Eng. 29 (5), 2977–3000. 10.1007/s11831-021-09685-5

[B15] Gray R. A. Pathmanathan P. (2018). Patient-specific cardiovascular computational modeling: Diversity of personalization and challenges. J. Cardiovasc. Transl. Res. 11 (2), 80–88. 10.1007/s12265-018-9792-2 29512059 PMC5908828

[B16] He Y. Northrup H. Le H. Cheung A. K. Berceli S. A. Shiu Y. T. (2022). Medical image-based computational fluid dynamics and fluid-structure interaction analysis in vascular diseases. Front. Bioeng. Biotechnol. 10, 855791. 10.3389/fbioe.2022.855791 35573253 PMC9091352

[B17] Hu J. Li F. Qiu P. Wu X. Pu H. Zhao Z. (2022). Clinical validation of the impact of branch stent extension on hemodynamics in ISF-TEVAR involving LSA reconstruction. Front. Cardiovasc. Med. 1448, 911934. 10.3389/fcvm.2022.911934 PMC9234204 35770224

[B18] Iqbal S. (2013). A comprehensive study of the anatomical variations of the circle of willis in adult human brains. J. Clin. diagnostic Res. JCDR 7 (11), 2423–2427. 10.7860/jcdr/2013/6580.3563 PMC3879841 24392362

[B19] Klimek-Piotrowska W. Rybicka M. Wojnarska A. Wojtowicz A. Koziej M. Holda M. K. (2016). A multitude of variations in the configuration of the circle of willis: An autopsy study. Anatomical Sci. Int. 91 (4), 325–333. 10.1007/s12565-015-0301-2 26439730

[B20] Lantz B. M. Foerster J. M. Link D. P. Holcroft J. W. (1981). Regional distribution of cardiac output: Normal values in man determined by video dilution technique. AJR. Am. J. Roentgenol. 137 (5), 903–907. 10.2214/ajr.137.5.903 7027775

[B21] Madhavan S. Kemmerling E. M. C. (2018). The effect of inlet and outlet boundary conditions in image-based CFD modeling of aortic flow. Biomed. Eng. online 17 (1), 66. 10.1186/s12938-018-0497-1 29843730 PMC5975715

[B22] Morbiducci U. Gallo D. Massai D. Consolo F. Ponzini R. Antiga L. (2010). Outflow conditions for image-based hemodynamic models of the carotid bifurcation: Implications for indicators of abnormal flow. J. Biomech. Eng. 132 (9), 091005. 10.1115/1.4001886 20815639

[B23] Morris P. D. Narracott A. von Tengg-Kobligk H. Silva Soto D. A. Hsiao S. Lungu A. (2016). Computational fluid dynamics modelling in cardiovascular medicine. Heart (British Card. Soc. 102 (1), 18–28. 10.1136/heartjnl-2015-308044 PMC4717410 26512019

[B24] Murray C. D. (1926). The physiological principle of minimum work: I. The vascular system and the cost of blood volume. Proc. Natl. Acad. Sci. U. S. A. 12 (3), 207–214. 10.1073/pnas.12.3.207 16576980 PMC1084489

[B25] Olufsen M. Peskin S. Kim W. Pedersen E. Nadim A. Larsen J. (2000). Numerical simulation and experimental validation of blood flow in arteries with structured-tree outflow conditions. Ann. Biomed. Eng. 28, 1281–1299. 10.1114/1.1326031 11212947

[B26] Pecoraro F. Lachat M. Cayne N. S. Pakeliani D. Rancic Z. Puippe G. (2017). Mid-term results of chimney and periscope grafts in supra-aortic branches in high risk patients. Eur. J. Vasc. Endovascular Surg. 54 (3), 295–302. 10.1016/j.ejvs.2017.06.014 28754428

[B27] Pirola S. Cheng Z. Jarral O. A. O'Regan D. P. Pepper J. R. Athanasiou T. (2017). On the choice of outlet boundary conditions for patient-specific analysis of aortic flow using computational fluid dynamics. J. Biomech. 60, 15–21. 10.1016/j.jbiomech.2017.06.005 28673664

[B28] Pirola S. Guo B. L. Menichini C. Saitta S. Fu W. G. Dong Z. H. (2019). 4-D flow MRI-based computational analysis of blood flow in patient-specific aortic dissection. IEEE Trans. Biomed. Eng. 66 (12), 3411–3419. 10.1109/Tbme.2019.2904885 30872222

[B29] Revellin R. Rousset F. Baud D. Bonjour J. (2009). Extension of Murray's law using a non-Newtonian model of blood flow. Theor. Biol. Med. Model. 6 (1), 7. 10.1186/1742-4682-6-7 19445663 PMC2695432

[B30] Romarowski R. M. Lefieux A. Morganti S. Veneziani A. Auricchio F. (2018). Patient-specific CFD modelling in the thoracic aorta with PC-MRI–based boundary conditions: A least-square three-element windkessel approach. Int. J. Numer. Method Biomed. Eng. 34 (11), e3134. 10.1002/cnm.3134 30062843

[B31] Scali S. T. Chang C. K. Feezor R. J. Hess P. J. Jr. Beaver T. M. Martin T. D. (2012). Preoperative prediction of mortality within 1 year after elective thoracic endovascular aortic aneurysm repair. J. Vasc. Surg. 56 (5), 1266–1273. 10.1016/j.jvs.2012.04.018 22840739 PMC4143903

[B32] Sengupta S. Hamady M. Xu X.-Y. (2022). Haemodynamic analysis of branched endografts for complex aortic arch repair. Bioengineering 9 (2), 45. 10.3390/bioengineering9020045 35200399 PMC8868591

[B33] Sesso H. D. Stampfer M. J. Rosner B. Hennekens C. H. Gaziano J. M. Manson J. E. (2000). Systolic and diastolic blood pressure, pulse pressure, and mean arterial pressure as predictors of cardiovascular disease risk in men. Hypertension 36(5): 801–807. 10.1161/01.HYP.36.5.801 11082146

[B34] Stergiopulos N. Westerhof B. E. Westerhof N. (1999). Total arterial inertance as the fourth element of the windkessel model. Am. J. Physiol. 276 (1-2), H81–H88. 10.1152/ajpheart.1999.276.1.h81 9887020

[B35] Stergiopulos N. Young D. F. Rogge T. R. (1992). Computer simulation of arterial flow with applications to arterial and aortic stenoses. J. Biomechanics 25 (12), 1477–1488. 10.1016/0021-9290(92)90060-E 1491023

[B36] Taylor C. A. Hughes T. J. Zarins C. K. (1998). Finite element modeling of three-dimensional pulsatile flow in the abdominal aorta: Relevance to atherosclerosis. Ann. Biomed. Eng. 26 (6), 975–987. 10.1114/1.140 9846936

[B37] Tricarico R. Laquian L. Allen M. B. Tran-Son-Tay R. Scali S. T. Lee T. C. (2020a). Temporal analysis of arch artery diameter and flow rate in patients undergoing aortic arch endograft procedures. Physiol. Meas. 41 (3), 035004. 10.1088/1361-6579/ab7b40 32109898

[B38] Tricarico R. Tran-Son-Tay R. Laquian L. Scali S. T. Lee T. C. Beck A. W. (2020b). Haemodynamics of different configurations of a left subclavian artery stent graft for thoracic endovascular aortic repair. Eur. J. Vasc. Endovasc. Surg. 59 (1), 7–15. 10.1016/j.ejvs.2019.06.028 31761570

[B39] van Bakel T. M. Arthurs C. J. van Herwaarden J. A. Moll F. L. Eagle K. A. Patel H. J. (2018). A computational analysis of different endograft designs for Zone 0 aortic arch repair. Eur. J. Cardiothorac. Surg. 54 (2), 389–396. 10.1093/ejcts/ezy068 29554234

[B40] Voskresensky I. Scali S. T. Feezor R. J. Fatima J. Giles K. A. Tricarico R. (2017). Outcomes of thoracic endovascular aortic repair using aortic arch chimney stents in high-risk patients. J. Vasc. Surg. 66 (1), 9–20.e3. 10.1016/j.jvs.2016.11.063 28216358 PMC5483394

[B41] Wallen T. J. Bavaria J. E. Vallabhajosyula P. (2018). Hybrid arch surgery challenges other forms of arch treatment. J. Cardiovasc. Surg. 59, 554–558. 10.23736/s0021-9509.18.10516-7 29687970

[B42] Westerhof N. Lankhaar J. W. Westerhof B. E. (2009). The arterial Windkessel. Med. Biol. Eng. Comput. 47 (2), 131–141. 10.1007/s11517-008-0359-2 18543011

[B43] Xu H. Piccinelli M. Leshnower B. G. Lefieux A. Taylor W. R. Veneziani A. (2018). Coupled morphological–hemodynamic computational analysis of type B aortic dissection: A longitudinal study. Ann. Biomed. Eng. 46 (7), 927–939. 10.1007/s10439-018-2012-z 29594688

[B44] Zhang J. Wang L. Chen Y. Wang S. Xing Y. Cui L. (2022). Color Doppler ultrasonography for the evaluation of subclavian artery stenosis. Front. Neurology 13, 804039. 10.3389/fneur.2022.804039 PMC8893015 35250811

