# Supplementary material for: Non-invasive estimation of the parameters of a three-element windkessel model of aortic arch arteries in patients undergoing thoracic endovascular aortic repair
Source: Front Bioeng Biotechnol. 2023 Feb 28;11:1127855. doi: 10.3389/fbioe.2023.1127855 (PMC10011467; doi:10.3389/fbioe.2023.1127855)
Supplement: Supplementary file 1 [file DataSheet1.pdf]

Supplemental Material

Demonstration that the normalized parameters do not alter the equation governing P-Q in a Windkessel model

With the following normalization scheme and definition

$$P_{norm} \stackrel{\text{def}}{=} \frac{P}{\bar{P}}; \quad Q_{norm} \stackrel{\text{def}}{=} \frac{Q}{\bar{Q}}; \quad R_{tot} \stackrel{\text{def}}{=} \frac{\bar{P}}{\bar{Q}};$$

$$R_{1norm} \stackrel{\text{def}}{=} \frac{R_1}{R_{tot}}; \quad R_{2norm} \stackrel{\text{def}}{=} \frac{R_2}{R_{tot}} = 1 - R_{1norm}; \quad C_{norm} \stackrel{\text{def}}{=} R_{tot} \cdot \frac{C}{T}; t_{norm} \stackrel{\text{def}}{=} \frac{t}{T}$$

Where $\bar{P}$ and $\bar{Q}$ are mean pressure and flow rate, respectively; T is the cardiac period. The non-normalized equation is: $\frac{\partial P}{\partial t} + \frac{P}{CR_2} = \frac{Q}{C}\left(1 + \frac{R_1}{R_2}\right) + R_1 \frac{\partial Q}{\partial t}$

The normalized equation is: $\frac{\partial P_{norm}}{\partial t_{norm}} + \frac{P_{norm}}{C_{\text{norm}} R_{2\text{norm}}} = \frac{Q_{norm}}{C_{\text{norm}}}\left(1 + \frac{R_{1\text{norm}}}{R_{2\text{norm}}}\right) + R_{1\text{norm}} \frac{\partial Q_{norm}}{\partial t_{norm}}$

By substituting the above definitions of P_{norm}, Q_{norm}, $R_{1\text{norm}}$, $R_{2\text{norm}}$, C_{norm}, and t_{norm},

$$\frac{\partial(\frac{P}{\bar{P}})}{\partial(\frac{t}{T})} + \frac{\frac{P}{\bar{P}}}{R_{tot} \frac{C}{T} \frac{R_2}{R_{tot}}} = \frac{\frac{Q}{\bar{Q}}}{R_{tot} \frac{C}{T}}\left(1 + \frac{\frac{R_1}{R_{tot}}}{\frac{R_2}{R_{tot}}}\right) + \frac{R_1}{R_{tot}} \frac{\partial(\frac{Q}{\bar{Q}})}{\partial(\frac{t}{T})}$$

$$\frac{\partial P}{\partial t} \frac{T}{\bar{P}} + \frac{P \cdot T}{\bar{P} C R_2} = \frac{Q \cdot T}{\bar{Q} R_{tot} C}\left(1 + \frac{R_1}{R_2}\right) + \frac{R_1 \cdot T}{R_{tot} \bar{Q}} \frac{\partial Q}{\partial t}$$

$$\frac{\partial P}{\partial t} + \frac{P}{CR_2} = \frac{\bar{P}}{\bar{Q}} \frac{Q}{R_{tot} C}\left(1 + \frac{R_1}{R_2}\right) + \frac{R_1}{R_{tot}} \frac{\bar{P}}{\bar{Q}} \frac{\partial Q}{\partial t}$$

$$\frac{\partial P}{\partial t} + \frac{P}{CR_2} = R_{tot} \frac{Q}{R_{tot} C}\left(1 + \frac{R_1}{R_2}\right) + \frac{R_1}{R_{tot}} R_{tot} \frac{\partial Q}{\partial t}$$

$$\frac{\partial P}{\partial t} + \frac{P}{CR_2} = \frac{Q}{C}\left(1 + \frac{R_1}{R_2}\right) + R_1 \frac{\partial Q}{\partial t}$$

which is the same as the non-normalized equation.

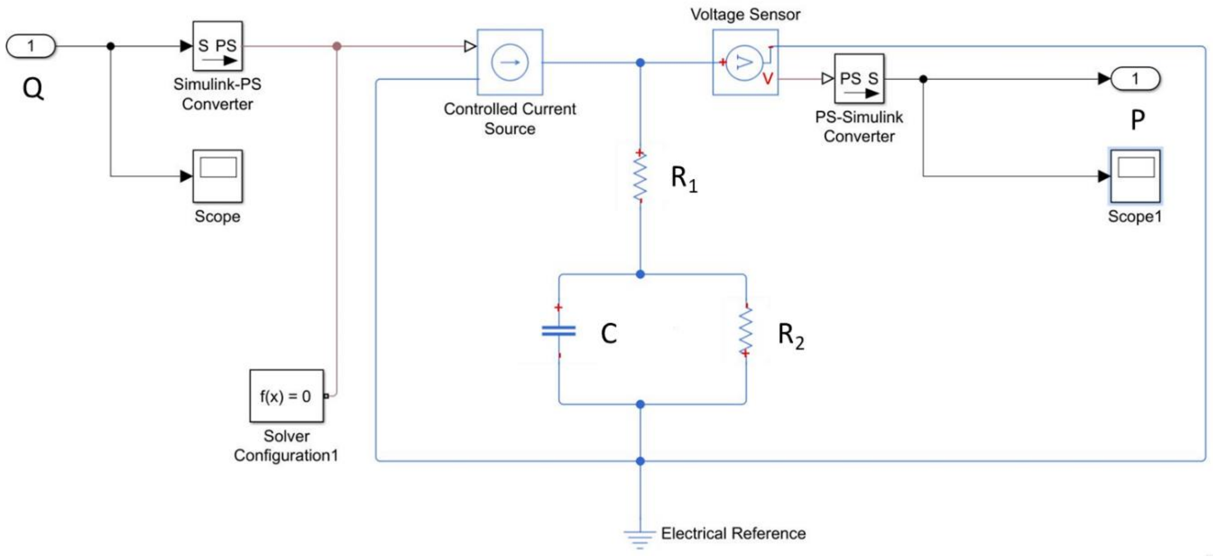

Supplemental Figure 1. The block diagram for the 3-element Windkessel model built in the Simulink Design Optimization toolbox. The resistor R_2 and the capacitor C, both in series to the resistor R_1, are connected to an electrical reference. The inlet flow rate (Q) and the expected outlet pressure (P) signals are imported to the system by the in-port and out-port, respectively. Two (inlet/outlet) scopes allow to display signals during the simulations. The Simulink-PS Converter block is needed to convert the input Simulink signal into a physical signal, and the PS-Simulink Converter block is used to convert the physical signal into a Simulink output signal. The Controlled Current Source block represents an ideal current source that is able to maintain the specified current through it regardless of the voltage across the source. The Voltage Sensor block represents an ideal voltage sensor, which is able to convert voltage measured between two points of an electrical circuit into a physical signal proportional to the voltage.

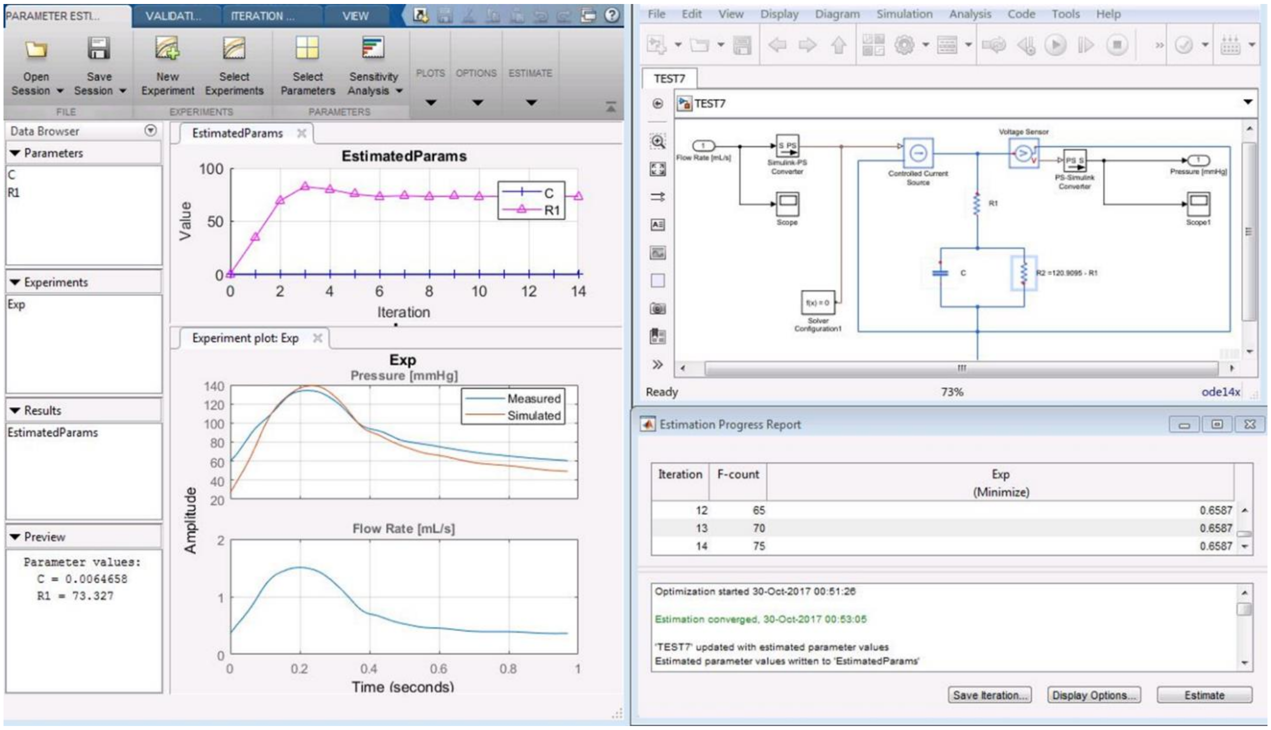

Supplemental Figure 2. The parameter estimation process. In the parameter estimation tool of the Design Optimization toolbox, R_1 and C were set up as the parameters to be estimated. The measured input and output signals were imported to the system using the New Experiment tool. The Runge-Kutta method was chosen as the solver with a fixed time step of 10^{-4} second. A nonlinear least square method and trust-region-reflective algorithm were utilized with both parameter tolerance and function tolerance of 0.001. The sum of squared error (SSE) between measured and simulated pressure of the WK3 model was chosen as the cost function for the optimization problem. In each iteration, the cost function quantified the quality of the pressure matching, and at the end of the optimization process, the optimized R_1, C, and R_2 combination was collected. Comparison between experimental and measured signals and parameter variations were plotted during the simulation. Additional details on how to use the Simulink Design Optimization toolbox for parameter estimation are available online on the MathWorks website (https://www.mathworks.com/products/sl-design-optimization.html).

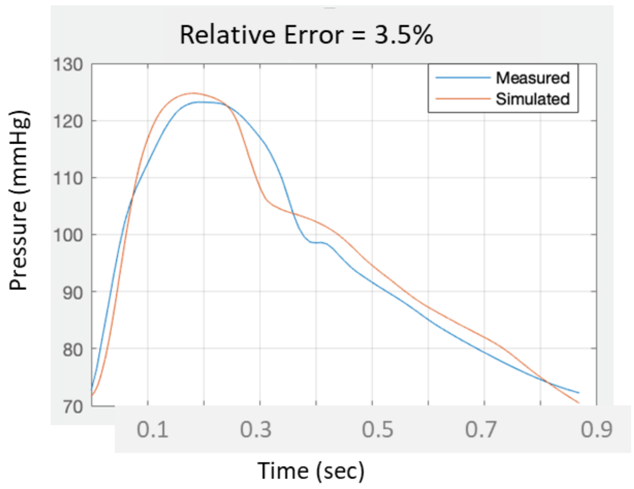

Supplemental Figure 3. One example of the comparison between the measured and simulated pressure waveforms with an overall relative error of 3.5%.

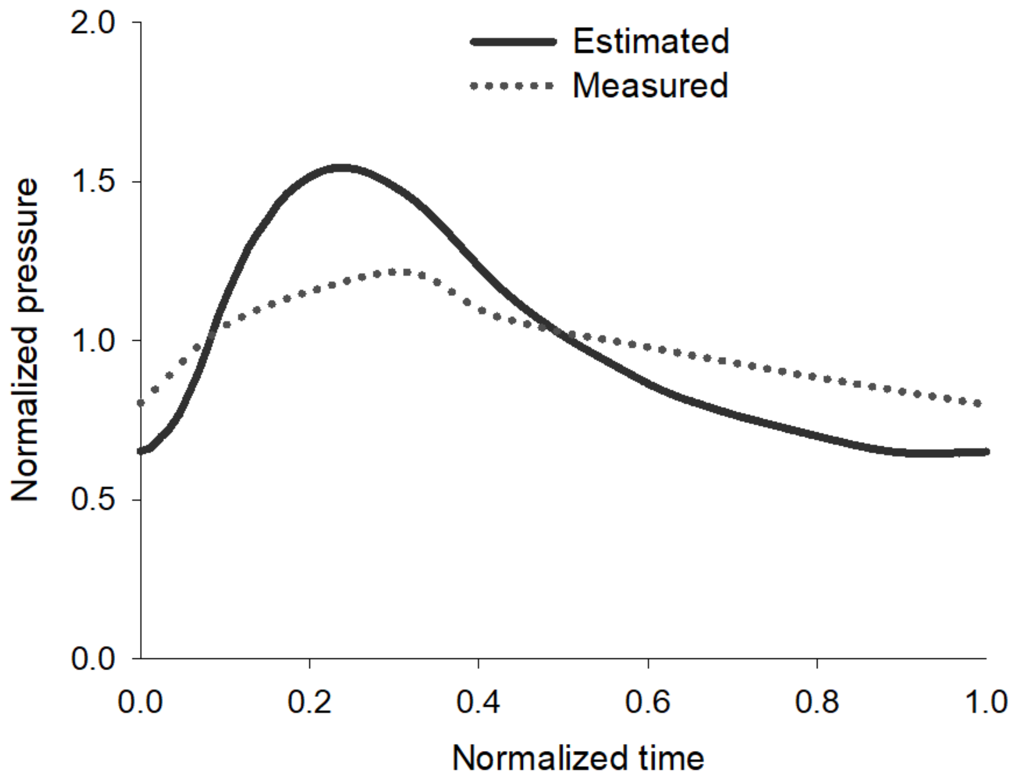

Supplemental Figure 4. One example of the measured and estimated pressure waveforms (normalized) of a subclavian artery when assessing the medians of normalized R_1, C, and R_2 parameters using the measured flow rate waveform as the input to the Windkessel model. There was a large difference between the two waveforms with a relative error of 18%. The flow rate waveform had a very high peak systolic and a large reversal diastolic flow (shown in Supplemental Figure 5).

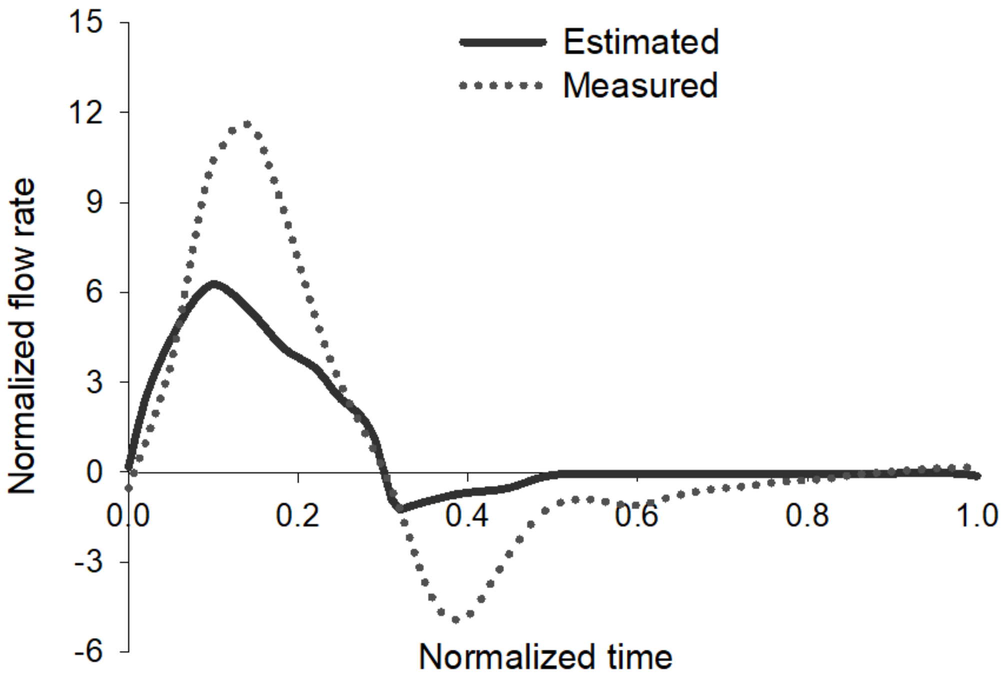

Supplemental Figure 5. One example of the measured and estimated flow waveforms (normalized) of a subclavian artery when assessing the medians of normalized R_1, C, and R_2 parameters using the measured pressure waveform as the input to the Windkessel model. There was a large difference between the two waveforms with a relative error of 94%. The measured flow rate waveform had a very high peak systolic and a high reversal diastolic flow.
